# Genetic and pharmacological tools to study the role of discoidin domain receptors in kidney disease

**DOI:** 10.3389/fphar.2022.1001122

**Published:** 2022-09-28

**Authors:** Corina M. Borza, Gema Bolas, Ambra Pozzi

**Affiliations:** ^1^ Department of Medicine (Division of Nephrology), Vanderbilt University School of Medicine, Nashville, TN, United States; ^2^ Veterans Affairs Hospitals, Nashville, TN, United States

**Keywords:** receptor tyrosine kinases, extracellular matrix, inhibitors, mouse models, cellular signaling, inflammation, acute and chronic injury

## Abstract

Following injury the kidney undergoes a repair process, which results in replacement of the injured tissue with little evidence of damage. However, repetitive injuries or inability of the kidney to stop the repair process result in abnormal deposition of extracellular matrix (ECM) components leading to fibrosis and organ dysfunction. The synthesis/degradation of ECM components is finely regulated by several factors, including discoidin domain receptors (DDRs). These are receptor tyrosine kinases that are activated by collagens. Upon activation, DDRs control several cell functions that, when exacerbated, contribute to kidney injury and fibrosis. DDRs are undetectable in healthy kidney, but become rapidly upregulated in several kidney fibrotic conditions, thus making them attractive anti-fibrotic targets. DDRs contribute to kidney injury and fibrosis by promoting apoptosis of injured kidney cells, stimulating the production of pro-inflammatory cytokines, and regulating the production of ECM components. They achieve these effects by activating canonical intracellular molecules or by directly interacting with nuclear chromatin and promoting the transcription of pro-fibrotic genes. The goal of this review is to highlight canonical and non-canonical mechanisms whereby DDRs contribute to kidney injury/fibrosis. This review will summarize key findings obtained using cells and mice lacking DDRs and it will discuss the discovery and development of targeted DDR small molecule- and antisense-based inhibitors. Understanding the molecular mechanisms whereby DDRs control kidney injury and fibrosis might enable us to not only develop more selective and potent inhibitors, but to also determine when DDR inhibition needs to be achieved to prevent and/or halt the development of kidney fibrosis.

## Discoidin domain receptor family members

Discoidin domain receptors (DDRs) belong to the family of transmembrane receptor tyrosine kinases (RTKs). This family includes two distinct members, DDR1 and DDR2 that specifically recognize collagens as their ligand. Both DDRs bind fibrillar collagens (e.g., collagens I-III); however for non-fibrillar collagens, DDR1 binds to basement membrane collagen IV while DDR2 binds collagen X (Shrivastava et al., 1997; Vogel et al., 1997; Hou et al., 2001; Leitinger et al., 2004; Leitinger and Kwan, 2006). Another characteristic of DDRs is that, unlike typical RTKs which are monomers in the absence of ligands, DDRs form stable ligand-independent noncovalent dimers that exist on the cell surface before ligand binding (Noordeen et al., 2006; Leitinger, 2014).

DDRs have three major domains: 1) an ectodomain that contains the N-terminal DS domain and a second globular domain, DS-like domain, that is unique to DDRs; 2) a single transmembrane domain that consist of an extracellular juxtamembrane region containing phosphorylatable tyrosine residues and a transmembrane helix mediating collagen independent receptor dimerization; and 3) an intracellular domain that is comprised of a large intracellular juxtamembrane region and a kinase domain followed by a short C-terminal tail (Carafoli and Hohenester, 2013; Iwai et al., 2014). Upon binding of collagen to the DS domain, DDRs undergo autophosphorylation of multiple tyrosine residues in the activation loop, which is likely to cause the kinase domain to switch from the inactive to the active state.

DDR2 has only 1 isoform, while DDR1 exists in five different isoforms (DDR1a–e) which are generated through alternative splicing (Alves et al., 2001). DDR1a–c encode functional receptor tyrosine kinases with DDR1b and DDR1c carrying an additional 37 amino acids within the intracellular juxtamembrane region, while DDR1d and DDR1e lack kinase activity. DDR1a and DDR1b are the major isoforms expressed in various tissues during development and in adulthood (Borza and Pozzi, 2014). DDRs regulate a number of fundamental biological processes including cell adhesion, migration, proliferation, and extracellular remodeling (Borza and Pozzi, 2014; Coelho et al., 2019; Auguste et al., 2020; Lafitte et al., 2020; Borza et al., 2022b). As mentioned above, the only difference between DDR1a and DDR1b is an extra 37 amino acids in the DDR1b intracellular juxtamembrane region preceding the kinase domain. Interestingly, these 37 extra amino acids contain an NPxY motif which we recently showed is critical for the binding of the scaffolding protein talin, localization of DDR1b to focal adhesions and promotion DDR1b-mediated cell migration (Borza et al., 2022b). With the exception of our and other few studies, the molecular mechanisms whereby DDRs exert their function are not fully understood. Proteomic analysis has been instrumental in generating a molecular signature of DDR signaling mechanisms in human health and disease and to advance our understanding of DDR biology (Lemeer et al., 2012; Iwai et al., 2013; Iwai et al., 2014). In addition, the generation of global as well as tissue selective DDR-null mice has enabled us to better understand the contribution of these RTKs to organ development and tissue function in both physiological and pathological setting. This review focuses on the contribution of DDR1 and DDR2 to kidney injury and disease.

## Discoidin domain receptor expression in disease

Abnormal DDR expression has been documented in pathological conditions including cancer, inflammatory and fibrotic diseases (Gao et al., 2021). In the context of kidney injury and disease, immunohistochemical analysis of DDR1 levels and localization showed that this receptor is upregulated in: 1) proximal tubule cells of subjects with transplant acute kidney injury (Chiusa et al., 2019); 2) cellular crescents as well as parietal epithelial cells and podocytes in glomerulonephritis (Kerroch et al., 2012; Moll et al., 2018); 3) parietal epithelial cells and distal tubule cells in Alport syndrome (Richter et al., 2019); and 4) tubule cells in diabetic nephropathy (Moll et al., 2019).

Consistent with the findings in subjects with kidney disease, DDR1 is upregulated in multiple animal models of kidney injury. In nephrotoxic serum-induced glomerulonephritis (NTS-SN), DDR1 is upregulated in podocytes, based on immunostaining (Kerroch et al., 2012) as well as in cellular crescents, parietal epithelial cells and tubular structures based on *in situ* hybridization (Moll et al., 2018). In hypertension-induced nephropathy, immunostaining revealed DDR1 upregulation in renal vessels and in glomeruli (Flamant et al., 2006), while in the unilateral ureteral obstruction (UUO) model, increased DDR1 expression is detected in the interstitium (Guerrot et al., 2011). In hereditary angiopathy with nephropathy, aneurysms, and muscle cramps syndrome DDR1 was shown to be upregulated in parietal epithelial cells by immunostaining (Chen et al., 2016), while in the remnant kidney model DDR1 is upregulated in glomeruli (Lee et al., 2004). Finally, western blot analysis revealed DDR1 upregulation in the mouse model of Alport syndrome (Richter et al., 2019). Most of the studies for DDR1 localization used a polyclonal antibody to DDR1 directed towards the C-terminus of DDR1. The specificity/cross-reactivity of this antibody has been recently questioned (Moll et al., 2019). Based on our experience, this antibody is highly cross-reactive in mouse tissue, with strong signals also in kidneys of injured DDR1-null mice. Thus, confirmation of DDR1 localization in injured mouse kidneys should be validated by the use of additional selective/validated anti-DDR1 antibodies.

Recently, using a model of severe ischemia/reperfusion-induced acute kidney injury (AKI) that progresses to chronic kidney disease (CKD) and tubulointerstitial fibrosis, we showed by Western blot analysis that DDR1 is upregulated and activated both in the acute and chronic phases of kidney injury (Borza et al., 2022a). A major hurdle to the analysis of DDR expression and localization in mouse models of kidney injury, is the lack of specific or validated anti-mouse DDR1 antibody. The availability of DDR1tm1a mice through EUCOMM (Coleman et al., 2015) that carry a LacZ gene between exons 5 and 6 of the DDR1 gene and have the LacZ gene regulated by the endogenous DDR1 promoter, can be used to localize DDR1 expression. We showed that DDR1tm1a have low and/or undetectable levels of β-galactosidase staining at baseline; however after severe AKI that progresses to CDK, a positive staining can be detected primarily in proximal tubules (the major tubules injured in AKI) at both acute and chronic phases of injury (Borza et al., 2022a). Ideally crossing these mice with genetic models of kidney diseases, or exposing these mice to various models of kidney injury can easily allow the analysis and visualization of renal DDR1 expression and localization at baseline and following injury.

The availability of mice lacking DDR1 has been instrumental in studying the role of this receptor in the initiation and progression of kidney disease. As summarized in Table 1, DDR1-null mice are protected from the development and/or progression of kidney disease following injury, clearly suggesting that upregulation of DDR1 observed in animal models of kidney injury and subjects with kidney disease contributes to kidney injury.

**TABLE 1:** Effect of DDR genetic deletion in mouse models of kidney disease.

Genetically deleted gene	Animal model	Outcome	Reference
DDR1	AngII-induced hypertensive nephropathy	Decreased periglomerular and interstitial fibrosis, decreased proteinuria, decreased macrophage number	Flamant et al. (2006)
DDR1	UUO	Reduced cytokine expression, reduced macrophage activation, reduced inflammation, reduced fibrosis	Guerrot et al. (2011)
DDR1	NTS-induced glomerulonephritis	Decreased proteinuria and uremia, reduced glomerular crescents, reduced fibrin deposits, reduced macrophage infiltration, reduced interstitial fibrosis	Kerroch et al. (2012)
DDR1	*Col4a3* ^−/−^ mice (Alport syndrome)	Increased survival, preserved renal function, decreased inflammation, decreased fibrosis	Gross et al. (2010)
DDR1	Partial renal ablation	Decreased proteinuria, reduced fibrosis	Borza et al. (2017)
DDR1	I/R with delayed nephrectomy	Decreased macrophage infiltration, reduced inflammation, reduced fibrosis	Borza et al. (2022a)
DDR2	UUO	Decreased fibrosis	Li et al. (2019b)

AngII, angiotensin II; UUO, unilateral ureteral obstruction NTS, nephrotoxic serum; I/R, ischemia/reperfusion.

In contrast to DDR1, whether and where DDR2 expression is upregulated in subjects with kidney disease has not been investigated. In some animal models of kidney injury like Alport syndrome (Sannomiya et al., 2021) and UUO (Li et al., 2019b), upregulation of DDR2 expression has been shown by Western blot and RT-PCR, respectively. However, no changes in DDR2 expression are observed in the remnant kidney model (Lee et al., 2004). However, whether increased expression contributes to disease seems to be disease model specific. To this end, mice lacking DDR2 show reduced renal interstitial fibrosis in the UUO model compared to injured wild-type mice (Li et al., 2019b) (Table 1). On the other hand, *in vivo* downregulation of DDR2 in Alport mice using DDR2-specific antisense oligonucleotide therapy did not improve proteinuria, nor it ameliorated renal injury, inflammation and fibrosis (Sannomiya et al., 2021) (Table 2). Thus, unlike DDR1 (Gross et al., 2010), DDR2 might not be critically involved in the pathogenesis of Alport syndrome.

**TABLE 2:** Targeting DDRs in mouse models of kidney disease.

Treatment	Animal model	Outcome	Reference
DDR1-specific antisense oligodeoxynucleotides	NTS-induced glomerulonephritis	Reduced podocyte injury, reduced inflammation, reduced fibrosis	Kerroch et al. (2012)
DDR1-specific antisense oligodeoxynucleotides	UUO NTS-induced glomerulonephritis	Preserved renal function, preserved renal structure	Kerroch et al. (2016)
DDR1 Small molecule inhibitor Roche-Chugai	NTS- induced glomerulonephritis NEP25-induced glomerulonephritis	Improved renal function, improved histology, decreased expression of inflammatory genes, decreased expression of fibrotic genes	Moll et al. (2018)
DDR1 Small molecule inhibitor 2.45	*Col4a3* ^−/−^ mice (Alport syndrome)	Preserved renal function, reduces renal fibrosis	Richter et al. (2019)
DDR2-specific antisense oligodeoxynucleotides	*Col4a3* ^−/−^ mice (Alport syndrome)	No beneficial effects on proteinuria, renal injury, inflammation or fibrosis	Sannomiya et al. (2021)

UUO, unilateral ureteral obstruction; NTS, nephrotoxic serum.

## Factors contributing to the regulation of discoidin domain receptor expression

Although it is well-established that the levels of DDRs are low/undetectable in healthy organs, changes in the expression of these two receptors are observed following injury or in disease. However, the mechanisms controlling DDR expression are not fully understood. Studies performed on cancer cells suggest that microRNAs negatively regulate DDR1 expression. To this end, lower levels of miR-199-3p correlate with increased DDR1 expression in ovarian cancer cells and in human ovarian cancer tissue (Deng et al., 2017) while decreased levels of miR-199-5p correlate with increased DDR1 expression in breast cancer cells (Mata et al., 2016). Moreover, in an animal model of cerebral ischemia injury the levels of miR-199a-5p are inversely correlated to DDR1 levels (Li et al., 2019a). Interestingly, in subjects with diabetic nephropathy there is a negative correlation between miR-199-3p levels and proteinuria, and renal tubular epithelial cells exposed to high glucose show a time-dependent decrease in miR-199-3p expression (Zhang et al., 2020). These findings, together with the evidence that increased expression of DDR1 is observed in the kidneys of subjects with diabetic nephropathy (Moll et al., 2019), seem to support a role for micro-RNAs in the control of DDR1 expression. In addition to micro-RNAs, cytokines have been shown to regulate the expression of DDR1. To this end, TGF-β upregulates DDR1 expression in hepatocarcinoma cells *via* SMAD-4 activation (Ezzoukhry et al., 2016). Interestingly, we showed that DDR1 promotes TGF-β production in renal proximal tubule cells *via* activation of signal transducer and activator of transcription 3 (Borza et al., 2022a), thus potentially creating a vicious cycle leading to upregulation of two major pro-fibrotic (TGF-β and DDR1) and pro-inflammatory (DDR1) molecules.

DDR1 expression can be negatively or positively regulated by various transcription factors in a cell type specific manner. The Zinc finger E-box-binding homeobox 1, for example, downregulates DDR1 expression in breast carcinoma cells (Koh et al., 2015) and in the neurons of spinal dorsal horn after treatment with the chemotherapeutic drug oxaliplatin (Chen et al., 2021). In contrast, the transcription factors YAP/TAZ associate with DDR1 promoter and upregulates its expression in vascular smooth muscle cells in response to substrate stiffness (Ngai et al., 2022).

Mechanisms that control DDR2 expression are less understood. Consistent with selective DDR2 mesenchymal expression, DDR2 is upregulated by the epithelial-to-mesenchymal inducing transcription factor TWIST1 in breast cancer cells (Grither et al., 2018; Lin et al., 2021). In cardiac fibroblasts, DDR2 expression is upregulated at both RNA and protein levels by the blood pressure controlling peptide hormone angiotensin II *via* activation of the transcription factor NF-κB (George et al., 2016). Interestingly, in the same cell type, DDR2 positively regulates the expression of the angiotensin receptor II expression and production of the pro-fibrotic molecule collagen I, thus creating a vicious cycle (Titus et al., 2021). In smooth muscles cells, the expression of DDR2 seems to be regulated by angiotensin II, TGF-β as well as by mechanical stretch (Jufri et al., 2015). To this end, smooth muscle cells exposed to cyclic mechanical stretch show increased DDR2 RNA and protein expression (Jufri et al., 2015). The finding that treatment of cells with p38MAPK small interfering RNA (siRNA), or the transcription factor c-myc siRNA prevents stretch-induced DDR2 upregulation suggests that intracellular kinases as well transcription factors can control DDR2 expression in the setting of mechanical forces (Jufri et al., 2015). As angiotensin II induces hypertension and elevated blood pressure that occurs with hypertension exposes cells to excessive mechanical load, together with the finding that angiotensin II controls DDR expression, the Angiotensin-II/DDR axis could represent a potential deleterious pathway activated in several diseases, including hypertension-mediated kidney disease.

## Regulation of discoidin domain receptor kinase activity

**FIGURE 1:**
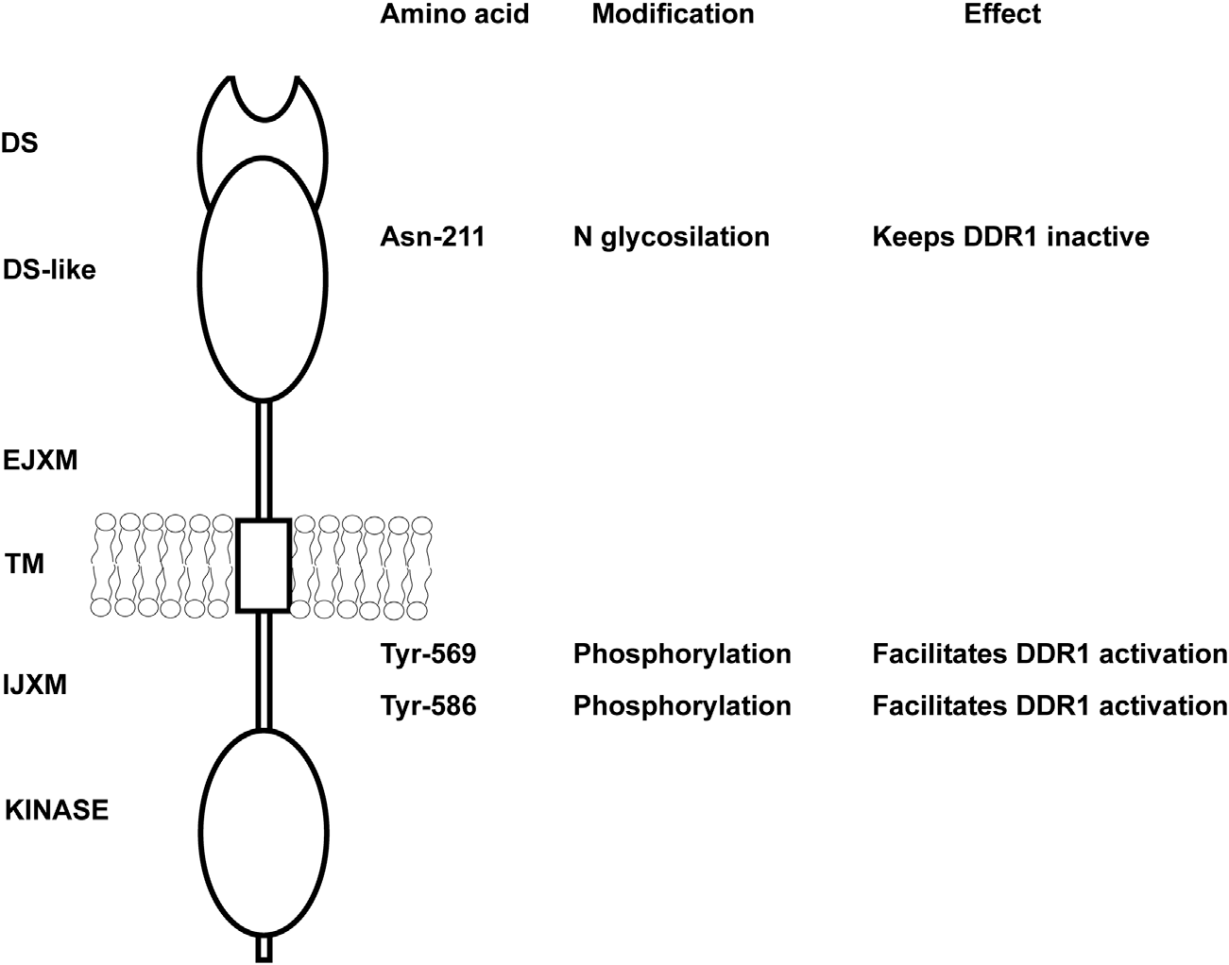
Key amino acids in DDR1 that regulate DDR1 activation. Schematic representation of DDR1 showing both extracellular and intracellular domains. N glycosylation of Asn-211 in the extracellular DS-like domain is key in keeping the receptor in an inactive, non-functional state. Mutation of this key amino acid results in collagen-independent constitutive phosphorylation of the receptor and prolonged DDR1 activation/phosphorylation following collagen treatment. Tyr-569 and Tyr-586 within the intracellular juxtamembrane domain are key in regulating DDR1 activation. Full activation of the DDR1 kinase in response to collagen requires first phosphorylation of Tyr-569 and Tyr-586, followed by autophosphorylation of key tyrosines in the kinase domain. DS, discoidin; EJXM, extracellular juxtamembrane; TM, transmembrane; IJXM, intracellular juxtamembrane.

As mentioned above, DDRs are unusual RTKs in that they form ligand-independent stable dimers that are noncovalently linked (Noordeen et al., 2006; Mihai et al., 2009) and undergo auto-phosphorylation upon binding to collagen. However, how collagen induces DDR phosphorylation or how the kinase activity of the receptors is regulated is not fully understood. DDR2, but not DDR1, requires the tyrosine kinase Src activity to be fully phosphorylated (Ikeda et al., 2002; Yang et al., 2005; Sammon et al., 2020). For DDR1, it has been proposed that upon collagen binding the receptor is activated in two distinct phases (Corcoran et al., 2019). In the first phase, which occurs rapidly within minutes, DDR1 redistributes into morphologically distinct clusters which contain unphosphorylated receptor. In the second phase, which is slower, DDR1 forms densely packed clusters which allow DDR1 phosphorylation between neighboring dimers (Juskaite et al., 2017). Whether, upon collagen treatment, a similar two-phase aggregation occurs for DDR2 phosphorylation is not known. An explanation for this low kinase activity of DDR1 has been suggested by Hanson et al. (2019) who used computational and biochemical studies to show that the DDR1 kinase domain is unusually stable in an inactive conformation. In addition, Sammon et al. (2020) showed that the intracellular juxtamembrane (JM) region JM4, proximal to the kinase domain, is critical for the autoinhibition of DDR1 kinase activity. They proposed that activation of the DDR1 kinase requires first phosphorylation of two tyrosine residues within the JM4, Tyr-569 and Tyr-586, followed by autophosphorylation of the tyrosines in the activation loop (Figure 1). However, phosphomimic mutation of Tyr-569 and Tyr-586 (capital T) in the full length DDR1—which should alleviate kinase autoinhibition thus resulting in an activated receptor—surprisingly results in a receptor that is no longer phosphorylated in response to collagen (Sammon et al., 2020). A possible explanation for this unexpected result, as suggested by the authors, is that the two tyrosine residues may play additional roles by interacting with DDR1 binding partners.

Another mechanism that controls DDR1 activation is glycosylation. N-glycosylation of the Asparagine 211 within the DS-like domain of DDR1 is important in negatively regulating DDR1 activation (Figure 1), as mutating this key amino acid results in collagen-independent constitutive phosphorylation of the receptor and prolonged DDR1 activation/phosphorylation following collagen treatment (Fu et al., 2014). Thus, glycosylation might be helpful in keeping the receptor in an inactive non-functional state. Overall, collagen-induced DDR activation is complex and requires collagen-induced aggregation and release from autoinhibition for DDR1 and participation of other kinases (e.g., Src) for DDR2.

## Cellular mechanism of discoidin domain receptor action

The mechanism whereby DDRs contributes to disease, particularly kidney disease, is not fully understood. Kidney disease is a multicellular process involving both resident and infiltrating cells that either directly or by interacting with each other can affect disease initiation and progression. Several studies indicate that DDR1 mediates in the initiation and development of inflammatory kidney disorders. However, whether DDR1 is expressed by immune cells thus directly regulating their function, or it stimulates an immune response by resident cells is still unclear. *In vitro* studies suggest that stimulated mononuclear cells of peripheral blood and T-cells express DDR1 which is required for cell migration in three-dimensional collagen matrices (Kamohara et al., 2001; Hachehouche et al., 2010; Chetoui et al., 2011). Mechanistically, in T-cells DDR1 promotes migration *via* the activation of RhoA/ROCK/MAPK/ERK signaling pathway (El Azreq et al., 2016; Kadiri et al., 2017). Similar to DDR1, DDR2 seems to be expressed on circulating human neutrophils and it regulates their migration in three-dimensional collagen matrices promoting chemotaxis through its ability to increase MMP-8 secretion (Afonso et al., 2013). DDR2 is also upregulated in collagen-mediated maturation of mouse bone marrow-derived dendritic cells (Lee et al., 2007) as well as in human monocyte-derived dendritic cells (Poudel et al., 2012). In dendritic cells, DDR2 regulates dendritic cell-mediated activation and proliferation of T-cells. DDR2 depletion significantly reduces dendritic cell-mediated production of inflammatory cytokine IL-2 and IFN-γ and T-cell proliferation (Lee et al., 2007; Poudel et al., 2012).

**FIGURE 2:**
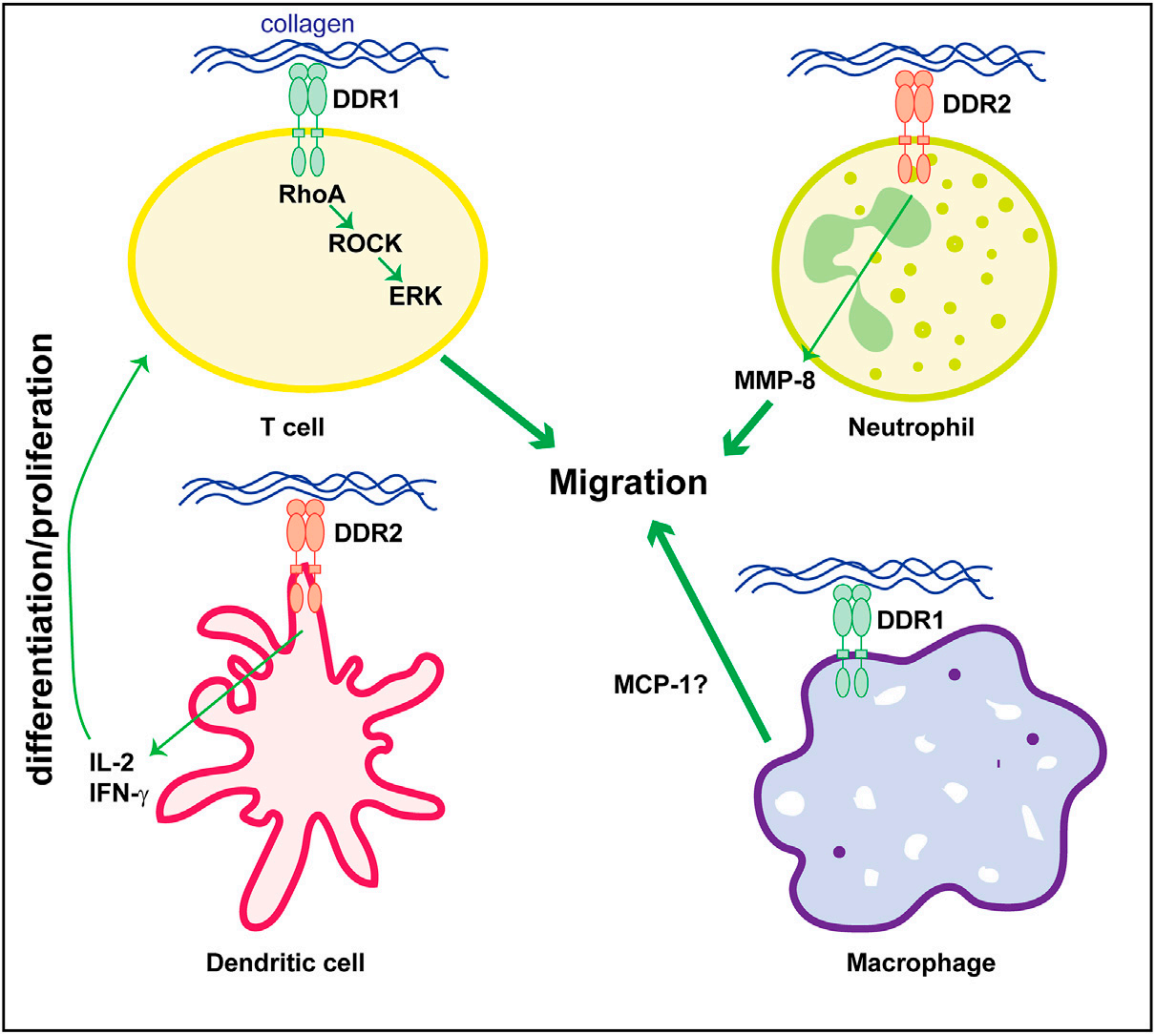
Role of DDRs in regulating immune cell responses. Collagen-mediated activation of DDR1 in T-cells promotes cell migration *via* activation of the RhoA/ROCK/ERK signaling. In neutrophils, cell migration is mediated by DDR2-induced secretion of MMP8 which promotes the generation of collagen-derived chemotactic peptide gradients. DDR2 expressed on dendritic cells promotes secretion of cytokines (e.g., IL-2 or IFN-γ) thus stimulating T-cell differentiation and/or proliferation. The direct role of DDR1 in macrophage is less clear. DDR1 seems to control cell migration by promoting secretion of MCP-1. However, this cytokine can also be produced by injured kidney cells thus indirectly promoting macrophage migration/infiltration. See text for details.

With the exception of these studies, very few groups have studied the expression and role of DDRs in immune cells particularly focused on kidney disease. Analysis of DDR1 expression by immunostaining, revealed increased expression of this collagen receptor on infiltrating macrophages in kidneys of mice subjected to UUO (Guerrot et al., 2011). Expression of DDR1 on macrophages seems to promote MCP1-induced cell migration, suggesting that DDR1 contributes to kidney damage by directly promoting inflammatory responses (Guerrot et al., 2011). In contrast to this finding, DDR1 is not found expressed in inflammatory cells in injured kidneys but it seems to promote immune cell infiltration *via* cytokine secretion by injured kidney cells (Flamant et al., 2006; Gross et al., 2010; Kerroch et al., 2012; Kerroch et al., 2016; Moll et al., 2019; Borza et al., 2022a). Figure 2 summarizes some of the functions exerted by DDR1 and DDR2 on immune cells.

**FIGURE 3:**
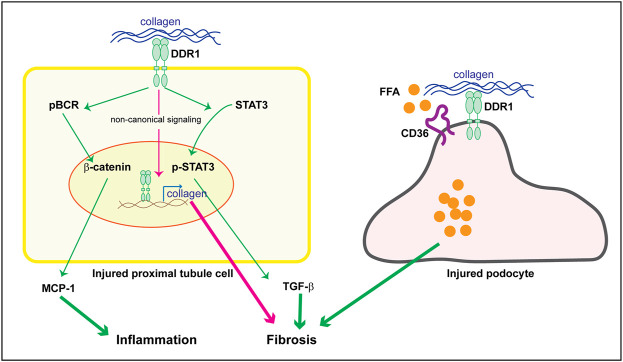
Potential DDR1-mediated pro-inflammatory and pro-fibrotic responses in resident kidney cells. Upregulation of DDR1 in injured proximal tubule cells can contribute to inflammation by promoting MCP-1 production *via* a BCR/β-catenin pathway. It addition, it can contribute to fibrosis by translocating to the nucleus and promoting collagen transcription (non canonical signaling) or TGF-β secretion *via* STAT3 activation. In injured podocytes, DDR1 can interact with CD36 thus leading to free fatty acid (FFA) uptake, cell damage and in turn fibrosis. See text for details.

As mentioned above staining performed on kidneys of subjects with human kidney disease as well as the use of DDR1tma1 mice, clearly show upregulation of DDR1 in injured resident cells, such as glomerular and/or tubular cells. These data suggest that DDR1 in resident cells might contribute to disease progression. DDR1 deleterious effects can be due to its ability to induce immune responses as well to stimulate pro-fibrotic responses in injured resident cells. We showed that DDR1 on renal proximal tubular epithelial cells promotes the production of the pro-inflammatory cytokine MCP-1 in response to collagen. Mechanistically, activation of DDR1 results in phosphorylation of its downstream target Breakpoint Cluster Region Protein (BCR) which results in β-catenin activation and in turn MCP-1 production (Borza et al., 2022a) (Figure 3). The observation that proximal tubule epithelial cells lacking DDR1 expression fail to phosphorylate BCR and promote MCP1 production in response to collagen, clearly indicates that DDR1/BCR is a key pathway involved in the promotion of immune responses by renal epithelial cells (Borza et al., 2022a). In addition to MCP1, DDR1 activation in pancreatic cancer cells initiates PKCθ/SYK/NF-κB signaling cascade which increases CXCL5 production and results in the recruitment of tumor associated neutrophils and formation of neutrophils extracellular traps (Deng et al., 2021). In adipose stem/progenitor cells, DDR1 promotes the secretion of IL6 (Sun et al., 2018). Interestingly, CXCL5 has been shown to drive neutrophil recruitment and kidney damage in a mouse model of TH17-mediated glomerulonephritis (Disteldorf et al., 2015), and IL6 is a cytokine shown to accelerate renal fibrosis after AKI *via* activation of β-catenin (Guo et al., 2022).

While the contribution of DDR1 in promoting the secretion of pro-inflammatory cytokines by kidney resident cells is well described, the role of DDR2 in promoting inflammatory responses by these cells is not clear. However, studies conducted in other organs or cell systems suggest that DDR2 can indeed stimulate the production of pro-inflammatory cytokines. To this end, human embryonic kidney cells overexpressing DDR2 increased secretion of IL-12 production *via* NF-κB and JNK pathway in response to collagen (Poudel et al., 2013). Moreover, in the synovial tissue of rheumatoid arthritis patients, DDR2 mRNA expression was significantly associated with the levels of pro-inflammatory IL-15, and collagen-induced activation of DDR2 in human fibroblast like synoviocytes results in increased IL-15 production thus promoting inflammation (Mu et al., 2020). Interestingly, angiotensin II—which is upregulated in many mouse models of kidney disease—can induce expression of DDR2 (George et al., 2016) and can promote secretion of IL-15 in primary renal parenchymal cells (Li et al., 2022). In the kidney, IL-15 promotes CD8^+^ tissue-resident-memory T-cell formation and activation, thereby promoting podocyte injury and glomerulosclerosis (Li et al., 2022). Thus both DDR1 and DDR2 can contribute to inflammation and kidney damage by promoting the secretion of pro-inflammatory cytokines by resident cells.

In addition to promoting the production of pro-inflammatory cytokines, DDRs can also contribute to disease progression by stimulating the production of extracellular matrix components. However, the pro-fibrotic actions of DDRs seems to be cell, tissue and organ specific. For example, mammary glands from DDR1-null mice contain substantially more collagen in the adipose tissue (Vogel et al., 2001) and DDR1-null mammary tumors contain significantly more fibrillar collagen than tumors expressing DDR1 (Takai et al., 2018). Moreover, deletion of DDR2 promotes carbon tetrachloride-induced hepatic fibrosis (Olaso et al., 2011). Although it is not clear how deletion of these receptors promotes collagen accumulation in breast and liver, a possible explanation is that loss of DDR1 could lead to upregulation of DDR2, or vice versa, as well as increased expression/activation of other pro-fibrotic matrix receptors such integrins. In contrast to these findings suggesting an anti-fibrotic action by DDRs, deletion of DDR1 or DDR2 protects the mice from bleomycin induced-lung injury or fibrosis (Avivi-Green et al., 2006; Zhao et al., 2016). In the context of kidney injury, collagen-induced activation of DDR1 initiates signaling pathways that promote fibrosis by directly increasing collagen production in resident cells as well as production of pro-fibrotic cytokines like TGF-β (Guerrot et al., 2011; Borza and Pozzi, 2014; Borza et al., 2017; Chiusa et al., 2019; Borza et al., 2022a) (Figure 3). We showed that in human renal proximal tubule cells, collagen-activated DDR1 translocates to the nucleus *via* interaction with nonmuscle myosin IIA and β-actin (Chiusa et al., 2019). In the nucleus, DDR1 binds to chromatin to promotes collagen IV transcription, a key collagen upregulated in kidney fibrosis (Chiusa et al., 2019). Interestingly, in mesenchymal cells overexpressing DDR1, activated DDR1 interacts with nonmuscle myosin IIA thus regulating fiber alignment and compaction, a key process that influences tissue fibrosis (Coelho et al., 2017). More recently, we showed that DDR1 activation in mouse proximal tubule epithelial cells promotes STAT3 phosphorylation and TGF-β production, a process that is inhibited by treatment of cells with a selective DDR1 ATP-competitive small molecule inhibitor (Borza et al., 2022a). By expressing various DDR1 mutants in mesangial cells lacking DDR1, we showed that DDR1-mediated collagen production requires a functional kinase domain, as mutations of DDR1 in the collagen binding site or in the kinase domain significantly reduce DDR1-mediated collagen production (Borza et al., 2017). Thus, in the lung and kidneys DDRs, particularly DDR1, seem to play a pro-fibrotic action, clearly suggesting that their function is highly tissue and organ dependent.

## Discoidin domain receptors and metabolism

It is well recognized that changes in lipid metabolism can lead to both inflammation and fibrosis. Excessive lipid accumulation or defective fatty acid oxidation is associated with increased lipotoxicity, which directly contributes to the development of fibrosis (Hwang and Chung, 2021). Because extracellular matrix mechanical cues regulate lipid metabolism (Romani et al., 2019) and mechanical stretch promotes DDR expression (Jufri et al., 2015), investigators have started to analyze the contribution of DDR-extracellular matrix interaction in lipid metabolism and disease. Mice lacking DDR1 together with the low-density lipoprotein receptor Ldlr (DDR1/Ldlr-null mice) show increased energy expenditure and brown fat activity, and overall reduced adipose tissue fibrosis compared to Ldlr-null mice (Lino et al., 2020). In the context of kidney injury, podocytes isolated from Alport mice show DDR1 activation in response to collagen that correlates with increased fatty acid uptake and triglyceride content (Kim et al., 2021). Mechanistically, activated DDR1 interacts with CD36 thus promoting lipid influx, lipotoxicity and cell damage (Figure 3). Treatment of Alport mice with the cholesterol lowering agent ezetimibe significantly decreased fibrosis in the Alport mice and protected podocytes from injury (Kim et al., 2021). Although this study seems to link DDR1 with lipid accumulation and cell damage, whether the *in vivo* effects are directly linked to DDR1 is not clear. Analysis of lipid accumulation in Alport mice treated with a selective DDR1 inhibitor, or crossed with DDR1-null mice would help in determining whether the reduced kidney injury due to lack of DDR1 activity indeed correlates to changes in lipid uptake by podocytes.

Although not directly investigated in kidney disease, increasing evidence implicates DDR2 in lipid physiology. Mice lacking DDR2 have reduced body mass index and adipose amount compared to wild type mice (Kawai et al., 2014). Moreover, selective deletion of DDR2 in adipose tissue enhances lipolysis *via* activation of the Adcy5-cAMP-PKA pathway (Yang et al., 2022). Thus, both DDR1 and DDR2 are positive regulators of fatty metabolism and they could contribute to disease by favoring fatty accumulation and increasing extracellular matrix deposition, thus creating a vicious cycle promoting fibrosis.

## Discoidin domain receptors and cell survival

Following AKI, the injured epithelia de-differentiate and proliferate resulting in repair (Ishibe and Cantley, 2008). When epithelial injury occurs repetitively or persists over time, tubular apoptosis may occur leading to severe renal injury. Increased DDR1 expression is observed in proximal tubules of mice subjected to AKI (Borza et al., 2022a) and subjects with transplant AKI (Chiusa et al., 2019). Because receptor tyrosine kinases regulate cell survival, it is reasonable to conceive that, in addition to regulating pro-inflammatory and pro-fibrotic signaling, DDR1 might be also involved in the regulation of proximal tubule cell survival. Although a direct role of DDRs in renal cell survival/apoptosis has not been evaluated, a dual pro- and anti-apoptotic role of DDRs has been shown in cancer progression (Mehta et al., 2021). DDR1 exerts a pro-survival, anti-apoptotic action in prostatic cancer, hepatocellular carcinoma and non-small-cell lung carcinoma (Shimada et al., 2008; Park et al., 2015; Villalba et al., 2019; Azizi et al., 2020). Mechanistically, DDR1 seems to promote cell survival by activating the Ras/Raf/MAPK pathway resulting in increased expression of the anti-apoptotic protein p53 (Ongusaha et al., 2003). In contrast to this finding, DDR1 is a pro-apoptotic receptor in breast cancer. In three-dimensional collagen matrices, DDR1 promotes apoptosis of breast carcinoma cells through induction of the pro-apoptotic Bcl-2-interacting killer protein (Maquoi et al., 2012; Saby et al., 2019). In addition, DDR1 promotes apoptosis of colon cancer cells which can be inhibited by the low density lipoprotein receptor related protein-1. When cultured in three-dimensional collagen matrices, lipoprotein receptor related protein-1 promotes DDR1 endocytosis thus decreasing cell apoptosis (Le et al., 2020).

In contrast to DDR1, DDR2 seems to have primarily pro-survival and anti-apoptotic function. In ovarian cancer, activation of DDR2 seems to enhance ovarian cancer cell survival by activating the Src-AKT pathway (Titus et al., 2021). Moreover, in cardiac fibroblasts, DDR2 confers resistance against oxidative stress by enhancing the expression of the antiapoptotic molecule cIAP2 *via* ERK1/2 MAPK-activated serum response factor (SRF) transcription factor pathway (Titus et al., 2020). Finally, fibroblasts lacking DDR2 expression are more prone to apoptosis, *in vitro* and *in vivo* due to inability to activate the PDK1 (3-phosphoinositide dependent protein kinase-1)/Akt survival pathway (Jia et al., 2018). Collectively, DDRs can exert both pro- and anti-apoptotic action and this seems to be dependent on the cell and disease type. Opposite DDR-mediated effects on cell survival and apoptosis could be due to different mechanisms, including: 1) selective cellular expression of DDR1 (mainly epithelial) vs. DDR2 (mainly mesenchymal); 2) selective ligand (e.g., fibrillar vs. non-fibrillar collagen)-mediated activation of the receptors that could lead to the initiation of cell specific intracellular signaling; and 3) DDR kinase dependent vs. independent activation of intracellular signaling.

## Pharmacological approaches for targeting discoidin domain receptors

Targeting DDRs in order to block their pro-inflammatory, pro-fibrotic or pro-tumorigenic effects is an attractive therapeutic option. There are several strategies that can be used to target DDRs including blocking their expression with antisense oligodeoxynucleotides (Kerroch et al., 2012; Kerroch et al., 2016), targeting the extracellular domain in order to prevent engagement with collagen (Grither and Longmore, 2018; Sun et al., 2021), or blocking their kinase activity (Kothiwale et al., 2015; Ambrogio et al., 2016; Aguilera et al., 2017; Hur et al., 2017; Jin et al., 2018).

In the context of kidney injury, downregulation of DDR1 expression with antisense oligodeoxynucleotides (AON) in the mouse model of NTS-GN preserves renal function and structure (Kerroch et al., 2012). Moreover, AON-induced downregulation of DDR1, immediately after the initiation of the disease protects mice from the progression of glomerulonephritis. In the UUO model, downregulation of DDR1 expression with AON 2 days after injury reduced inflammation and preserved renal structure (Kerroch et al., 2016). In contrast to this finding, downregulation of DDR2 expression with AON in a mouse model of Alport syndrome did not decrease proteinuria, inflammation or fibrosis (Sannomiya et al., 2021), despite lowering the levels of MCP-1 and collagen I. It is not clear whether lack of overall beneficial effects are due to incomplete depletion of DDR2 or whether DDR2 does not play a role in the kidney injury in this animal model. In addition, due to the lack of validated mouse DDR2 antibody, it is not clear which cells in the kidneys express this collagen receptor. Crossing the Alport mice with DDR2-null mice would help in answering some of these outstanding questions. Although AON seems to be a promising approach in dampening DDR1-mediated deleterious effects in mouse models of kidney diseases, whether downregulating DDR1 expression is a feasible treatment option for patients with kidney disease remains to be determined. Table 2 summarizes the effects of AON-mediated DDR1 targeting in mouse models of kidney disease.

Targeting the extracellular domain of DDRs was shown to be beneficial in animal models of cancer. A recent study that looked at the role of DDR1 in triple-negative breast cancer showed that DDR1 inhibits the infiltration of anti-tumor immune cells by promoting collagen fibers alignment (Sun et al., 2021). This protective function only requires the extracellular domain, but not the kinase domain of DDR1. Antibodies directed against the DDR1 extracellular domain inhibited DDR1-mediated collagen fibers alignment and tumor growth (Sun et al., 2021). Similar to DDR1, blocking DDR2 interaction with collagen using the small molecule inhibitor WRG-28 resulted in profound inhibition of metastatic breast tumor cell colonization in the lungs (Grither and Longmore, 2018). Because some of the DDR2-mediated effects on tumor invasion, migration and metastasis are independent of its kinase activity (Barcus et al., 2021), targeting DDR2-collagen interaction represents a promising options to halt tumor growth and invasion. Whether preventing DDR/collagen interaction can be applied also in the context of kidney disease is unclear. We and others have shown that in the kidney, DDR1 pro-inflammatory or pro-fibrotic effects require a functional kinase activity of the receptor. Thus, inhibition of DDR1 kinase activity seems to be an alternative attractive option.

Since blocking DDRs kinase activity emerged as an attractive therapeutic option in cancer, inflammatory, and fibrotic diseases (Bansod et al., 2021; Denny and Flanagan, 2021; Elkamhawy et al., 2021; Dong et al., 2022), significant effort has been made to identify potent and selective DDR1 inhibitors. DDR kinase inhibitors are largely ATP-competitive inhibitors that can be classified as type I or type II inhibitors. Type I inhibitors bind the kinase in the active conformation with the highly conserved DFG-motif of the activation loop in DFG-Asp-in conformation. Type II inhibitors bind to the kinase in the inactive conformation with DFG motif facing away from the active site (DFG-Asp-out) (Kothiwale et al., 2015). However, the DFG-Asp-out conformation is unusually stable in DDR1 and facilitates promiscuous inhibitor binding (Hanson et al., 2019). Surprisingly, DDR1 has been shown to bind type I inhibitors, like dasatinib, in an inactive conformation (Hanson et al., 2019).

Several potent DDRs inhibitors have been described which showed efficacy in blocking DDR-mediated biological effects in cell culture systems or *in vivo* animal models. Most of the DDR kinase inhibitors so far developed have been studied primarily in the context of cancer. For instance, the DDR1 inhibitor 3-(2-(pyrazolo[1,5-a] pyrimidin-6-yl) ethynyl) benzamide (7rh) significantly reduced tumor growth in gastric cancer xenografts (Hur et al., 2017), tumor burden in KRAS-driven lung adenocarcinoma (Ambrogio et al., 2016) and reduced primary tumor burden and improved chemoresponse in pancreatic ductal adenocarcinoma (Aguilera et al., 2017). Thus, DDR kinase inhibitors have been generated in the last decade and clearly show efficient inhibition of DDR1-mediated biological effects in animal models of cancer. Whether these type of inhibitors can be used in kidney disease, would require a careful analysis of their selectivity, toxicity and pharmacokinetic properties.

We reported the discovery of a dual potent and selective DDR1/2 inhibitor, VU6015929, which blocks collagen-induced DDR1 activation and collagen IV production in mesangial cells while displaying low cytotoxicity and acceptable *in vitro* DMPK profile (Jeffries et al., 2020). Another dual DDR1/2 inhibitor, XBLJ-13, significantly and dose-dependently inhibits lung inflammation and fibrosis in the bleomycin-induced pulmonary fibrosis animal model (Dong et al., 2022). Two studies targeting the kinase activity of DDR1 in animal models of kidney disease have been reported. The first study (Moll et al., 2018) used an inhibitor developed by Roche-Chugai in two models of glomerulonephritis: 1) nephrotoxic-serum -induced glomerulonephritis and 2) NEP-25-glomerulonephritis, a mouse model in which human CD25 is expressed in podocyte and can be targeted with LMB2 (immunotoxin binding to human CD25 thus resulting in podocyte injury and development of GN (Matsusaka et al., 2005). For the NTS-GN injury model the inhibitor was used at a low dose (1x coverage of IC50, 75 mg/kg) and high dose (10X coverage of IC50, 200 mg/kg), in a prophylactic regime. In the NEP-25-GN the inhibitor was administered at low dose (50 mg/kg) starting 7 days after the LMB2 treatment. Interestingly, in the NTS-GN model the DDR1 inhibitor at high, but not at low dose preserved renal function and reduced tubulo-interstitial inflammation and fibrosis. The effect of the DDR1 inhibitor in the NEP-25 models was modest most likely due to its low dose and therapeutic regime. These results seem to suggest that complete DDR1 inhibition is required to achieve the desired protective biological effect. Due to the low dose used, whether DDR1 inhibitors can be used as prophylactic or therapeutic drugs in kidney disease, needs to be better evaluated.

The second study used a DNA-encoded library-derived DDR1 inhibitor in a mouse model of Alport syndrome. The DDR1 inhibitor used, 2.45, has good selectivity, metabolic stability, pharmacokinetic and physicochemical properties (Richter et al., 2019). When administered intraperitoneally, daily, at 90 mg/kg for 4 weeks, starting at 4 weeks of age, the DDR1 inhibitor preserved renal function and reduced tissue damage. As the experiment was stopped at week 8 is not clear whether this DDR1 inhibitor, would increase survival of Alport mice as observed in the Alport mice crossed with the DDR1-null mouse (Gross et al., 2010). Moreover, is not clear at what time point DDR1 is overexpressed in the Alport mice and whether administering the inhibitor when the receptor starts to be expressed would provide better therapeutic benefits. Overall, these two studies showing some beneficial effects of blocking DDR1 in the context of kidney disease, establish DDR1 as a promising therapeutic target. However, further studies in the optimization of DDR1 inhibitors, target engagement and evaluation of potential off target effects is required.

## Conclusion

DDRs are involved in the initiation and progressions of several diseases, including cancer and fibrotic diseases. In the context of kidney disease, cytokines and peptide hormones such as TGF-β and angiotensin-II that are either upregulated in or contribute to kidney disease might exert their deleterious effects by promoting the expression of DDRs (Ezzoukhry et al., 2016; George et al., 2016). In turn, DDRs could contribute to disease progression by activating apoptotic, inflammatory and fibrotic signaling in a cell type dependent manner. Unlike other tyrosine kinases receptors, DDRs are highly expressed only and/or primarily in injured cells, thus making them an ideal and selective therapeutic target. Several strategies aimed to target DDRs in acute and chronic mouse models of kidney disease have been developed and tested with promising results. Inhibition of DDRs in mice seems to be well-tolerated with no overall and/or reported side effects; however, more studies are needed to better define the prophylactic versus therapeutic effects of DDR inhibition in kidney disease.

Although DDR expression is upregulated in subjects with acute and chronic kidney disease (Chiusa et al., 2019), identification of patient populations that would benefit from a DDR targeted therapy would definitely facilitate bench-to-bedside transition. Interestingly, the extracellular domain of DDR1 can be cleaved by membrane bound matrix metalloproteinases (Fu et al., 2013) and analysis of serum levels of cleaved DDR1 in subjects affected by liver fibrosis or cirrhosis showed that levels of cleaved DDR1 represented a powerful diagnostic tool and an accurate biomarker that associates with the severity of liver fibrosis (Zhang et al., 2021). Thus, analysis of cleaved DDR1 in the urine and/or plasma of subjects affected by kidney disease may enable the identification of fast progressors who could benefit from anti-DDR selective therapy.
